# Clocks and Heart Disease

**Published:** 1895-09-07

**Authors:** 


### Clocks and Heart Disease.

By a ready transposition of an old adage it has been said that punctuality is the thief of time, and many *insouciant* individuals who lounge through life with but small consideration for the people they keep waiting are ready to assert of their more punctual friends that the time they waste in maintaining their character for punctuality would make up for many a delay by which much profit might be gained. This is a question we will not enter into, and we may frankly admit that occasions may arise when, notwithstanding the old saying that delays are dangerous, it may be excusable not to keep an appointment to the minute. While, however, we may excuse an occasional lapse in so wayward and irregular an animal as man, among whose virtues punctuality after all is but one, the matter is otherwise in regard to clocks. In them punctuality is their first and single duty, and want of it a vice for which neither grace, nor tunefulness, nor beauty can atone. It would be difficult to say how many people are annually brought to the grave by ill-timed clocks, but it is no small number, and if we consider also the immensity of the worry and anxiety produced by the same cause we shall not hesitate to welcome the suggestion which is being put into practical effect by the municipality of Glasgow that public clocks should be made to synchronise so that no one need further hesitate as to what is the time of day. This matter has a very distinctly medical bearing, for it is to the sudden runs and frantic rushes to which middle-aged people are so often urged by the irregularity of public clocks that the onset of obvious heart disease is too frequently due. We do not suggest that running to catch a train is at all commonly a cause of injury to a healthy heart; but who among our city men above the age of fifty has a healthy heart, with a muscle so sound that it can carry him without injury through a sharp three minutes' run, ending with a clamber up a steep flight of steps to a railway platform, while he is laden with the black bag of the suburban dweller in the one hand, an umbrella and overcoat in the other, and perhaps a bundle of books as well? Hundreds of men are going about leading useful, happy, and fairly active lives, yet whose hearts have lost the elasticity of youth, and can bear no strain without irreparable injury. By an unconscious adaptation to circumstances the habits of these men have become so attuned to the power of their hearts that the growing weakness of that organ is not perceived. In fact, the heart may not be at all too weak for the sort of life the owner leads. "I'm not delicate" each one would in all honesty protest, for the chain is not felt till the end of its tether is attained; and so when some ill-regulated clock, respectable in appearance and with nothing to show the untruth of the lies that it proclaims, suggest to him that he will miss his train, he runs, and perhaps while he runs he does not feel particularly distressed, but when he reaches the carriage, panting, palpitating, and exhausted, he knows he has done too much, and although it is only at long intervals that such a man actually dies in the attempt, doctors know well how often the final run to catch a train picks out the weak organ and is the starting point of a long, slow, and most miserable process of failure from dilated heart, ending in dropsy and in death.

A pernicious custom has grown up of putting the clock outside some railway stations a little in advance of the real time. If this were done uniformly everyone would soon get to know about it, and, although it would be silly and absolutely useless, it still might not do much harm. As it is the harm is infinite. The rush and turmoil of railway travelling is added to by the uncertainty as to the real time, and many a man who might have toddled on comfortably to a comparatively old age is cut off in the midst of his work and usefulness by the lying suggestions of a clock which whispers to him, "You will miss your train!"

We would urge then that the necessary complement, even if it be not the proper antecedent, to the work of the Glasgow municipality in providing official clocks would be the compulsory removal of all clocks which tell either more or less than the true time.

